# Hepatitis C subtype distribution in chronically infected patients with mild liver fibrosis in France: the GEMHEP study

**DOI:** 10.1017/S0950268819001225

**Published:** 2019-07-10

**Authors:** T. Semenova, B. Nemoz, V. Thibault, G. Lagathu, G. Duverlie, E. Brochot, P. Trimoulet, C. Payan, S. Vallet, C. Henquell, S. Chevaliez, M. Bouvier-Alias, S. Maylin, A-M. Roque-Afonso, L. Izquierdo, F. Lunel-Fabiani, P. Marcellin, P. Morand, V. Leroy, S. Larrat

**Affiliations:** 1 Laboratoire de Virologie, Institut de Biologie et Pathologie, CHU Grenoble-Alpes, Grenoble, France; 2 Laboratoire de Virologie, CHU de Rennes, Rennes, France; 3 Laboratoire de Virologie- EA4294 Centre de Biologie Humaine CHU Amiens, Université de Picardie Jules Verne, Amiens, France; 4 Laboratoire de Virologie, CHU de Bordeaux, Bordeaux, France; 5 Département de Bactério-Virologie, Hygiène Hospitalière et Parasito-Mycologie, CHRU La Cavale Blanche, Brest, France; 6 Service de Virologie, CHU de Clermont-Ferrand, Clermont-Ferrand, France; 7 Département de Virologie, Bactériologie-Hygiène, Mycologie-Parasitologie, Unité Transversale de Traitement des Infections, Créteil, France & INSERM U955, Créteil, France; 8 Hôpital Saint Louis, Service de Microbiologie- Pôle B2P, Paris, France; 9 Laboratoire de Virologie, Hôpitaux Universitaires Paris-Sud, Hôpital Paul Brousse, Villejuif, France; 10 Laboratoire de Virologie, CHU Angers, HIFIH laboratory, UPRES EA 3859, SFR 4208, Angers, France; 11 Service d'Hépatologie, Hôpital Beaujon, Clichy, France; 12 Clinique d'Hépato-gastroentérologie, Pôle Digidune, CHU Grenoble-Alpes, Grenoble, France

**Keywords:** Epidemiology, fibrosis, HCV genotypes, treatment-naive HCV

## Abstract

Treatment options for Hepatitis C infection have greatly improved with direct-acting antiviral (DAA) combinations achieving high cure rates. Nevertheless, the cost of this treatment is still high and access to treatment in many countries has been preferentially reserved for patients with more severe fibrosis (F3 and F4). In this French nationwide study, we investigated the epidemiological characteristics and genotype distribution of hepatitis C virus (HCV) in treatment-naive patients with METAVIR fibrosis stages between F0 and F2 in order to identify patient profiles that became eligible for unrestricted treatment in a second period. Between 2015 and 2016 we collected data from nine French university hospitals on a total of 584 HCV positive patients with absent, mild or moderate liver fibrosis. The most represented genotypes were genotype 1b (159/584; 27.2%), followed by genotype 1a (150/584; 25.7%); genotype 3 (87/584: 14.9%); genotype 4 (80/584; 13.7%). Among genotype 4: 4a was predominantly encountered with 22 patients (27.5% of genotype 4). Genotypes 1b and 1a are currently the most frequent virus types present in treatment-naive patients with mild fibrosis in France. They can be readily cured using the available DAA. Nevertheless, non-a/non-d genotype 4 is also frequent in this population and clinical data on the efficacy of DAA on these subtypes is missing. The GEMHEP is the French group for study and evaluation of viral hepatitis on a national scale. Data collection on epidemiological and molecular aspects of viral hepatitis is performed on a regular basis in all main French teaching hospitals and serves as a basis for surveillance of these infections. Analysis and trends are regularly published on behalf of the GEMHEP group. Data collection was performed retrospectively over the 2015–2016 period, covering nine main university hospitals in France. A total of 584 hepatitis C positive patients were included in this study. Genotyping of the circulating viruses showed a high prevalence of genotypes 1b and 1a in our population. The epidemiology of hepatitis C is slowly changing in France, particularly as a consequence of the rise of ‘non-a non-d’ genotype 4 viruses mainly originating from African populations. More data concerning treatment efficacy of these genotypes is needed in order to guide clinical care.

## Introduction

Hepatitis C virus (HCV) infection is still a major public health concern, as shown by alarming epidemiological data. HCV prevalence is highly variable from one country to another, from 0.1% to 24% of the population, with 71.1 million viraemic people worldwide [1, 2]. According to the French National Agency for HIV and viral hepatitis (ANRS), 192 700 people live with HCV in France and 59% of them are not aware of their HCV status [3]. The introduction of direct-acting antiviral interferon-free treatment since 2014 has dramatically improved the rates of sustained viral response (SVR). For example, a combination of sofosbuvir and anti-NS5A treatment given to genotype 1-infected patients was associated with SVR rates from 92% to 99% in real life studies [4, 5]. However, the high cost of these new anti-HCV drugs has for long limited access to antiviral treatment for patients with mild to moderate fibrosis scores (METAVIR F0–F2). In order to limit costs for the French healthcare system, only patients with specific indications such as severe fibrosis, previous treatment failure or HIV infection and after individualised medical review were eligible for public funding of treatment [6, 7]. This strategy left many patients with mild fibrosis (F0–F2) treatment-naïve. Since March 2017, public funding criteria have been extended and this F0–F2 population is now eligible for antiviral therapy. In France, a large number of HCV patients, estimated at 150 000 according to the French health ministry, remain to be treated [2, 3, 8].

Knowledge of the various hepatitis C genotypes infecting treatment-naïve patients and their relative prevalence has important clinical implications. The aim of this study was to investigate the epidemiological characteristics and distribution of HCV genotypes in treatment-naive patients with a METAVIR fibrosis stage between F0 and F2, in France [3, 9].

## Patients and methods

### Patients

Data were collected from nine French tertiary hospitals (Hepato-Gastroenterology Departments and Laboratories) between 2015 and 2016. All patients were selected anonymously and had signed an informed consent form for data collection. The selection was according to the following criteria: absent, mild or moderate fibrosis; absence of HCV treatment, HCV viral load results available, sequencing and genotyping performed since 1 January 2015; and frozen serum samples available in case of need to repeat analyses. Exclusion criteria were: past or current HCV treatment and/or liver fibrosis >stage F2. Relevant characteristics including sex, age, mode of HCV acquisition, invasive and non-invasive methods used for liver fibrosis assessment (liver biopsy, FibroScan^®^, FibroMeter^®^, FibroTest^®^, ActiTest^®^), serum alanine aminotransferase (ALT) level, comorbidities (HIV or HBV coinfection, alcohol consumption) and sequencing methods (NS5B, NS3, Core/E1, innoLIPA) were collected for each patient. Other characteristics including country of origin or transmission route were recorded when known. The degree of liver fibrosis was evaluated using the FibroTest-ActiTest and/or transient elastography performed using a FibroScan (Echosens, Paris, France) device. Liver fibrosis was graded on the METAVIR scale from 1 to 4 [10]. Cut-off values of liver stiffness measurement by FibroScan were applied as previously described [11]: 2.5–7 kPa absent or early fibrosis (F0–F1); 7–9 kPa mild fibrosis (*F* = 2), 9–12 kPa severe fibrosis (*F* = 3) and >12 kPa cirrhosis (*F* = 4). When two fibrosis tests were available the most severe fibrosis score was attributed as recommended by the French association for the study of Liver disease (AFEF) [7]. When the fibrosis score was between F0 and F1, or between F1 and F2, it was classified as ‘F1’ and ‘F2’ respectively. Serum ALT levels were determined locally using various manufacturers' assays. Results were expressed as a multiple of the upper limit of normal (ULN). Plasma viral load was measured in each centre using CE approved assays according to the manufacturers' instructions and results were expressed in IU/ml.

### HCV genotyping and phylogenetic analyses

HCV genotyping was performed locally using NS5B, NS3 and core sequencing or the Line Probe Assay v2.0 (InnoLiPA). Five laboratories used only NS5B sequencing techniques, three laboratories used 2 genotyping assays (NS5B + core, NS5B + InnoLiPA, NS5B and NS3) and one laboratory used all three sequencing methods. Genotyping was determined according to a previously described algorithm [12–14]. All sequencing data were collected and analysed in a unique central laboratory. All sequences were divided into three groups according to the genomic region (core, NS5B, NS3) and aligned using a MAFFT (Multiple alignment program for amino acid or nucleotide sequences) (mafft.cbrc.jp/alignment/software) and a standard algorithm *FFT-NS-2-I* [15, 16]. Phylogenetic analysis was visualised by the neighbour-joining method with 1000 bootstraps [17]. The first tree containing all sequences was constructed for crude genotype determination using selected reference sequences obtained from Genbank. Next, specific trees were built for each genotype to assess the subtype. The genotyping results from the participant laboratories were compared to those from the centralised phylogenetic analysis.

### Statistical analyses

Demographic characteristics are expressed as mean ± standard deviation (s.d.), as median with interquartile range or as percentages. To compare categorical and nonparametric data we used the *χ*^2^ test and Mann–Whitney or Kruskal–Wallis tests, respectively. Statistical significance was considered at P < 0.05. Statistical analyses were conducted using R (Version 0.99.893; RStudio, 2016).

Univariate and multivariate analyses were performed using the logistic regression function. The variables at the univariate analysis with significant association were analysed by multivariate analysis. Statistical significance of the logistic regression function was considered at 5%.

## Results

### Characteristics of treatment-naïve patients with HCV infection in France

**Fig. 1.**
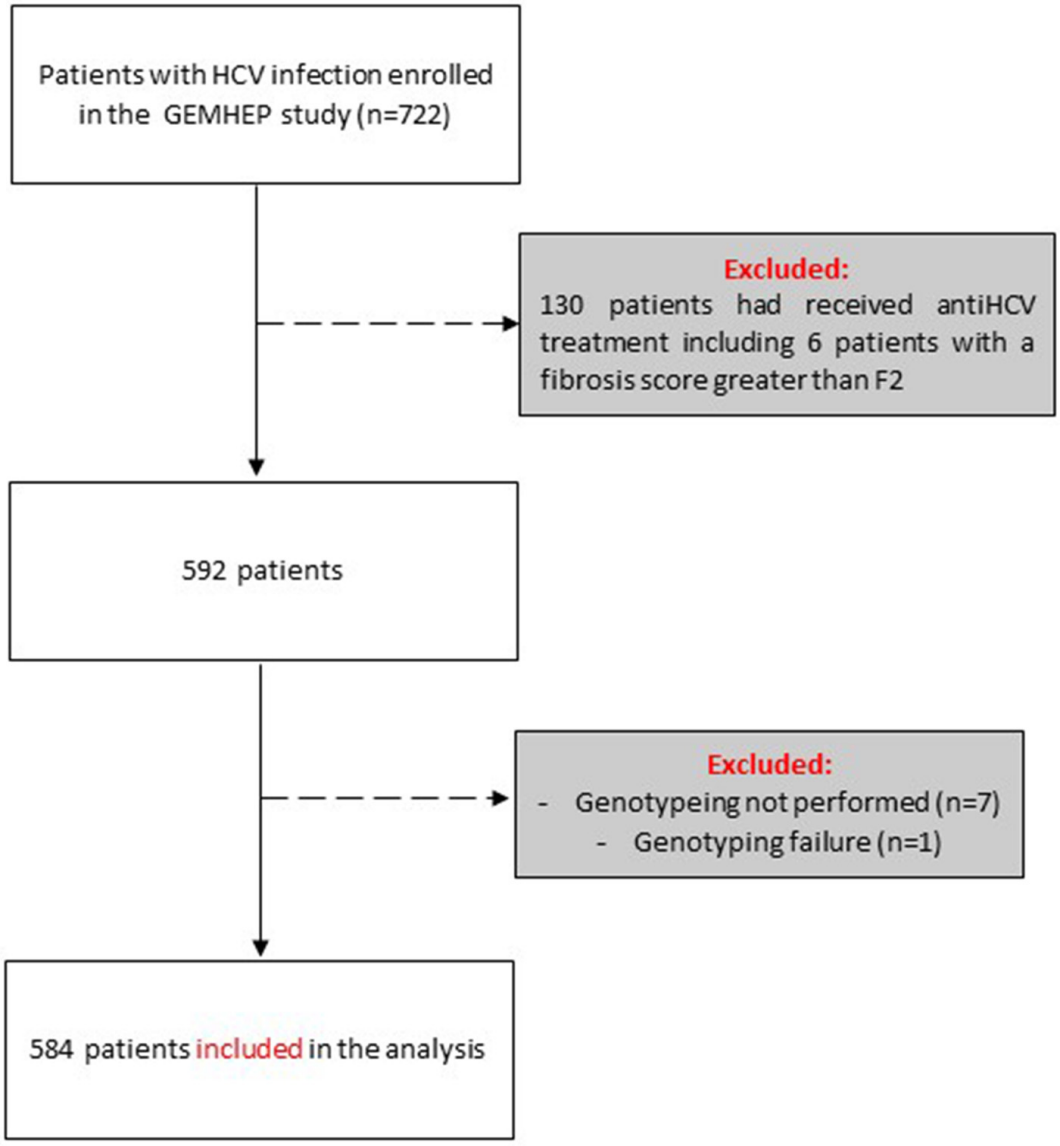
Flow chart of patient inclusion in the GEMHEP study. Patients excluded from the study are indicated in grey boxes.

**Fig. 2.**
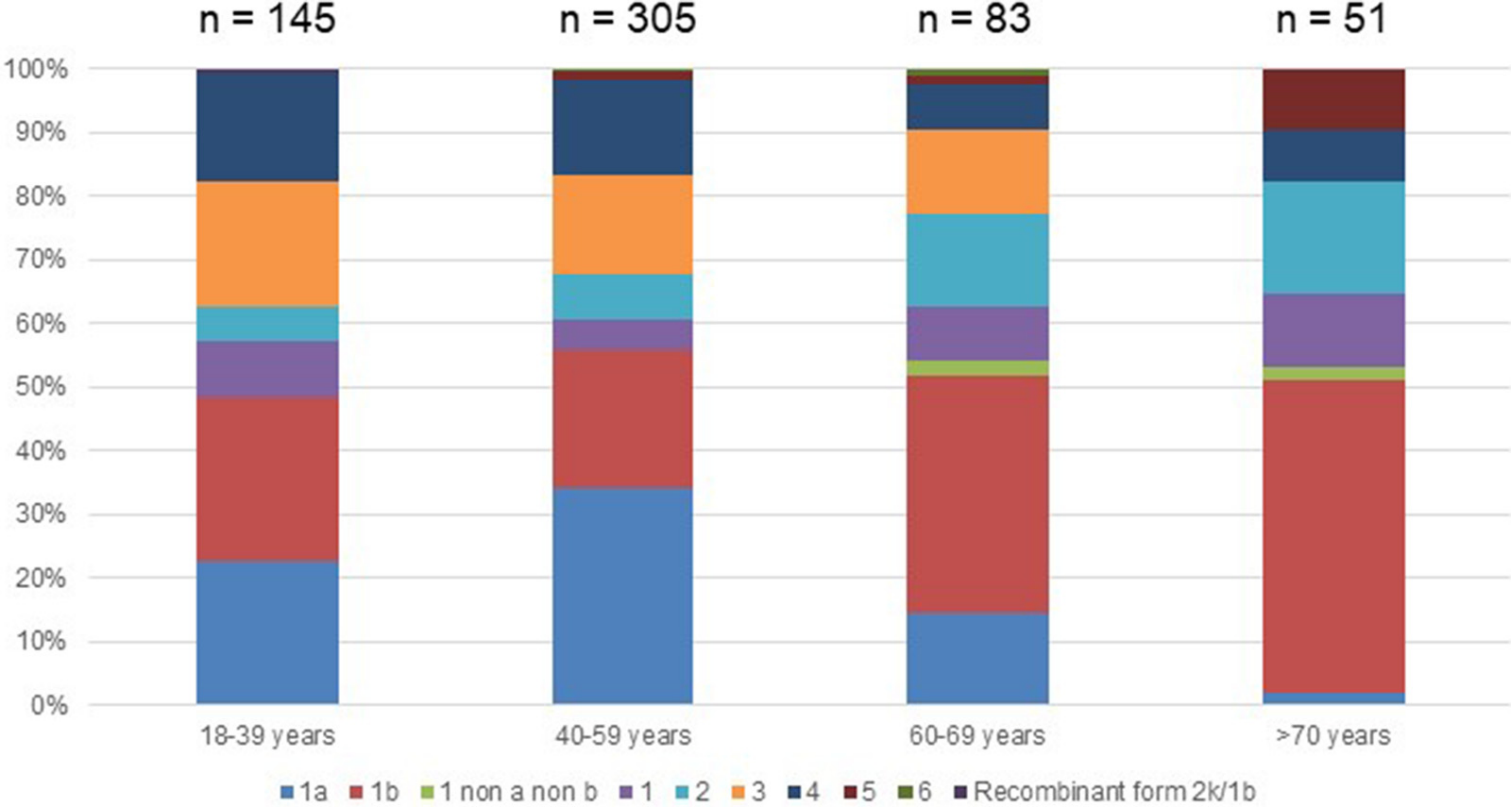
Age distribution of Hepatitis C virus genotypes in treatment naïve patients with fibrosis stage F0–F2 in France,%.

Out of the 722 patients initially selected, 138 patients presented at least one exclusion criterion (Fig. 1). The demographic characteristics of the remaining 584 analysed patients are shown in Table 1. We found a significant difference in HCV infection frequency between men and women (47% *vs.* 53%, *P* = 0.02). The 40–59-year-old group was the most frequently infected (34%) (Fig. 2).

**Table 1.** Characteristics of untreated patients with hepatitis C and fibrosis stage F0–F2 included in the French GEMHEP study

Characteristics	Data
Age, median (interquartile range)	50 (39–58)
Age group, *N* (%)	
18–39 years	145 (25)
40–59 years	305 (52)
60–69 years	83 (14)
>70 years	51 (9)
Sex, *N* (%)	
Male	276 (47)
Female	308 (53)
Duration of HCV infection (years^a^) Data available for 193 patients	27.6 (±12.8)
Serum HCV RNA (log 10 IU/ml^a^) Data available for 245 patients	5.9 (±0.75)
Serum ALT (x Normal)	1.5 (±1.2)
HIV co-infection, *N* (%) Data available for 338 patients	30 (9)
HBV co-infection, *N* (%) Data available for 338 patients	6 (2)
Chronic alcohol intoxication (%) Data available for 338 patients	96 (28)
Activity stage by ActiTest, *N* (%) Data available for 306 patients	
A0	83 (27)
A1	127 (42)
A2	64 (21)
A3	32 (10)
Fibrosis stage by Fibrotest/FibroMeter or Fibroscan, *N* (%) Data available for 584 patients	
F0	160 (28)
F1	328 (56)
F2	96 (16)

N/A, not available.

a Mean, ±s.d.

### Distribution of HCV genotypes in treatment-naïve patients in France

As shown in Table 2, 353 (60.5%) patients harboured a virus of genotype 1. The HCV genotype 1b was predominant, followed by genotypes 1a, 3, 4, 2, 5 and genotype 6 (Supplementary Fig. S1). The recombinant form 2k/1b was found in one patient. The genotype of this last sample appeared dubious on the phylogenetic tree but was confirmed using near-full-length HCV genome sequencing as described by Trémeaux *et al*. [18]. Genotype 2 was divided into several subtypes without any of them being clearly predominant. In contrast, the ‘a’ subtype was the unique genotype 3 subtype detected in our French HCV population. Among genotype 4 subtypes: 4a was found in 22 patients (27.5% of genotype 4) and 4d in 15 patients (18.8%) but we note that the majority (53.8%; *n* = 43) were classified as ‘non-a non-d’. Among them, we found 7 subtypes 4r, 6 4f, 3 4c, 3 4k.

**Table 2.** Hepatitis C virus genotype distribution in untreated patients with fibrosis stage between F0 and F2 in France

	1a	1b	1 non a non b	1 non subtyped	2	3	4	5	6	Recombinan*t* form 2k/1b	N (%)	*P*-value
Number, abs (%)	150 (25.68)	159 (27.23)	3 (0.51)	41 (7.02)	50 (8.56)	87 (14.9)	80 (13.71)	10 (1.71)	3(0.51)	1 (0.17)	584 (100)	0.02
Age, median (±s.d.)	47 (±11)	53 (±16)	69 (±8)	50 (±17)	54 (±15)	46 (±12)	46 (±13)	67 (±15)	52 (±14)	32		<0.0001
18–39 years, *N* (%)	33(22.8)	37 (25.5)		13 (9.0)	8 (5.5)	28 (19.3)	25 (17.2)			1 (0.7)	145 (100)	
40–59 years, *N* (%)	104 (34.1)	66 (21.6)		15 (4.9)	21 (6.9)	48 (115.7)	45 (14.8)	4 (1.3)	2 (0.7)		305 (100)	
60–69 years, *N* (%)	12 (14.5)	31 (37.3)	2 (2.4)	7 (8.4)	12 (14.5)	11 (13.3)	6 (7.2)	1 (1.2)	1 (1.2)		83 (100)	
>70 years, *N* (%)	1 (2.0)	25 (49.0)	1 (2.0)	6 (11.8)	9 (17.6)		4 (7.8)	5 (9.8)			51(100)	
Men (%)/women	83 (55)/67	60 (38)/99	1/2	14(34)/27	27(54)/23	49(56)/38	38(47)/42	3/7	0/3	1/0	276/308	0.004
ALT (x normal^a^)	1.6 (±1.6)	1.1 (±0.9)	1.1 (±0.7)		1.4 (±1.2)	2.5 (±0.9)	1.3 (±1.2)	0.7	1.35 (±0.2)	0.8		0.01
HCV Viral load, IU/ml												
<8 00 000 IU/ml (%)	26 (17.4)	55 (36.9)	1 (0.7)	N/A	6 (4.0)	32 (21.5)	24 (16.1)	3 (2.0)	1 (0.7)	1 (0.7)	149 (100)	
>8 00 000 IU/ml (%)	43 (28.1)	44 (28.8)	2 (1.3)	N/A	15 (9.8)	19 (12.4)	23 (15.0)	5 (3.3)	2 (1.3)		153 (100)	
⩾60 00 000 IU/ml (%)	12 (41.4)	5 (17.2)		N/A	4 (13.8)	6 (20.7)	2 (6.9)				29 (100)	
Fibrosis stage by Fibrotest/Fibrometer or FibroScan, (%)												<0.001
F0	27.5	24.4		6.3	8	15.0	15.6	1.9	1.3			
F1	25.0	28.0	0.9	7.9	8.8	14.9	13.1	0.6	0.5	0.3		
F2	25.0	29.2		5.2	8.3	14.6	12.5	5.2				

N/A, not available.

a Mean, ±s.d.

Genotype 1a and genotype 3 were more frequently found in men than in women (55% *vs.* 45% (*P* < 0.01) and 56% *vs.* 44% (*P* = 0.004), respectively) (Supplementary Fig. S2). Conversely genotype 1b predominated in women (38% *vs.* 62% (*P* < 0.001]). For genotypes 2 to 5 we found no significant difference in distribution between the sexes. The small number of genotype 6 included did not allow to conclude on any sex difference. Genotype distribution also varied according to age group (Fig. 2). Genotype 1a was mostly observed in the 18–39 and 40–59 age groups (22.8% and 34.1%, respectively). Genotype 1b was more frequently observed in older age groups: 60–69s and the over 70s (37.3% and 49.0% respectively). Mean age was significantly higher for patients infected with genotype 1b than those infected with genotype 1a (53 ± 16 and 47 ± 11 respectively, *P* < 0.0001).

In multivariate analyses, females in this cohort were significantly older than men and higher fibrosis scores were associated with older age (*P* = 0.014 and *P* < 0.0001, respectively). No relation between HCV genotype and any other demographic characteristics was observed.

Genotype distribution according to investigator centre was significantly different (*P* < 0.0001) and is shown in Supplementary Figure 3. The HCV RNA viral load was significantly different between HCV genotype groups. Genotypes 1a and 2 had higher HCV RNA concentrations compared to the other groups (*P* < 0.001). HCV RNA levels below 8 00 000 IU/ml were observed most frequently in patients with genotypes 1b (36.9%) and 3 (21.5%). In 29 patients the HCV RNA concentration was high (⩾60 00 000 IU/ml), including in 12 patients with genotype 1a (41.4%) (Table 2). We found that HCV high viral loads (> 8 00 000 UI/ml and >60 00 000 UI/ml) were significantly associated with genotypes 1a and 2 (OR 7.772, 95 CI 4.80–12.57; *P* < 0.0001 and OR 7.648, 95 CI 3.22–18.14, *P* < 0.0001, respectively) (Table 3). Multivariate analysis confirmed this association (Table 4).

**Table 3.** Univariate analysis of baseline factors for specific HCV genotypes infection

	1a		1b		1 non a non b		1x		2		3		4		5		6	
	OR (95 CI)	*P*	OR (95 CI)	*P*	OR (95 CI)	*P*	OR (95 CI)	*P*	OR (95 CI)	*P*	OR (95 CI)	*P*	OR (95 CI)	*P*	OR (95 CI)	*P*	OR (95 CI)	*P*
18–39 years	0.902 (0.01–84.75)	0.002	1.006 (0.01–94.09)	0.998	0.01 (0.005–2.123)	0.092	0.034 (0.01–3.27)	0.147	0.189 (0.02–18.67)	0.477	0.743 (0.08–70.94)	0.898	0.640 (0.07–60.43)	0.848	0.010 (0.01–2.11)	0.092	0.010 (0.01–125.47)	0.092
40–59 years	1.534 (0.02–142.65)	0.034	0.852 (0.01–79.05)	0.945	0.005 (0.02–0.99)	0.05	0.018 (0.03–1.67)	0.082	0.238 (0.02–22.79)	0.538	0.565 (0.06–53.55)	0.806	0.518 (0.06–48.44)	0.776	0.044 (0.01–4.51)	0.186	0.025 (0.01–2.12)	0.122
60–69 years	0.540 (0.01–51.95)	0.070	1.771 (0.02–166.77)	0.805	0.095 (0.01–10.43)	0.326	0.034 (0.01–3.30)	0.147	0.495 (0.05–48.44)	0.764	0.495 (0.05–48.47)	0.764	0.258 (0.03–25.65)	0.564	0.056 (0.01–6.85)	0.240	0.056 (0.01–2.69)	0.241
>70 years	0.097 (0.01–11.94)	0.903	3.389 (0.03–323.56)	0.600	0.097 (0.01–12.01)	0.343	0.018 (0.01–2.02)	0.096	0.749 (0.08–74.25)	0.902	0.032 (0.01–6.67)	0.207	0.310 (0.03–31.88)	0.621	0.388 (0.04–39.2)	0.688	0.032 (0.01–6.65)	0.205
Men	0.646 (0.44–0.93)	0.022	1.70 (1.17–2.47)	0.005	1.797 (0.16–19.93)	0.633	1.798 (0.92–3.50)	0.085	0.744 (0.41–1.13)	0.319	0.652 (0.41–1.03)	0.068	0.989 (0.62–1.58)	0.963	2.116 (0.54–8.26)	0.281	6.336 (0.32–123.85)	0.224
ALT	1.017 (0.95–1.08)	0.611	0.611 (0.46–0.80)	<0.0001	N/A	N/A	0.505 (0.03–0.87)	0.015	0.916 (0.67–1.25)	0.579	1.476 (1.21–1.78)	<0.001	0.832 (0.62–1.11)	0.213	N/A	N/A	0.918 (0.27–3.15)	0.892
HCV Viral load																		
<8 00 000 IU/ml	(Reference)																	
>8 00 000 IU/ml	7.772 (4.80–12.57)	<0.0001	0.296 (0.19–0.44)	<0.0001	1.753 (0.23–13.42)	0.589	N/A	N/A	10.657 (4.14–27.38)	<0.0001	0.266 (0.15–0.46)	<0.001	0.409 (0.24–0.68)	0.001	1.048 (0.31–3.47)	0.938	1.753 (0.23–13.42)	0.589
⩾60 00 000 IU/ml	7.648 (3.22–18.14)	<0.0001	0.318 (0.11–0.86)	0.025	3.194 (0.12–84.09)	0.487	N/A	N/A	4.144 (0.76–22.46)	0.099	0.931 (0.36–2.41)	0.883	0.311 (0.07–1.35)	0.120	0.858 (0.04–16.74)	0.919	3.194 (0.12–84.09)	0.487
Fibrosis stage																		
F0	(Reference)																	
F1	0.879 (0.57–1.34)	0.554	1.209 (0.784–1.87)	0.390	3.452 (0.17–67.81)	0.415	1.291 (0.60–2.75)	0.507	1.097 (0.55–2.17)	0.791	0.995 (0.58–1.69)	0.986	0.815 (0.47–1.38)	0.452	0.321 (0.05–1.94)	0.216	0.290 (0.04–2.23)	0.234
F2	0.879 (0.49–1.55)	0.661	1.278 (0.72–2.25)	0.399	1.663 (0.03–85.89)	0.800	0.824 (0.27–2.48)	0.732	1.028 (0.41–2.57)	0.953	0.967 (0.47–1.97)	0.928	0.771 (0.36–1.61)	0.492	2.875 (0.67–12.31)	0.155	0.328 (0.02–7.018)	0.476

OR, Odds Ratio; 95 CI, 95% Confident Intervals; *P*, *P*-value; N/A, not available due to small number.

**Table 4.** Multivariate analysis of baseline factors for HCV genotypes 1a and 2

	1a		2	
	OR (95CI)	*P*	OR (95CI)	*P*
>8 00 000 IU/ml	110 175 (6.722–18.057)	<0.0001	20.963 (8.058–54.533)	<0.0001
⩾60 00 000 IU/ml	8639 (3.561–20.964)	<0.0001	N/M	N/M

OR, odds ratio; 95 CI, 95% confident intervals; *P*: *P*-value; N/M, not measurable.

Data on the mode of HCV acquisition (nosocomial, intravenous drug use (IVDU) and other modes of HCV transmission) was available for 287 patients. For 119 patients identified as probable intravenous drug users (IVDU) genotypes 1a (42%) and 3a (22.6%) were the most predominant. Of the 87 patients infected by transfusion of blood products: 27 (31%) were genotype 1b, 17 (19.5%) were genotype 2 and 13 were genotype 1a (15%). The mode of HCV acquisition was only available for 34 genotype 4-infected patients, of whom 12 were contaminated via IVDU (genotype 4a and 4d). Among ‘non-a non-d’ genotype 4, the most common mode of HCV acquisition was not clearly defined but was neither nosocomial nor IVDU.

### Severity of liver disease and HCV genotypes

Mean serum ALT concentration was significantly higher in patients with genotype 1a than in other groups (*P* = 0.01), although the standard deviation for ALT was larger than in other genotypes. Table 1 shows data on necroinflammatory activity and fibrosis stage measured by FibroTest^®^ or FibroMeter^®^. The fibrosis stage varied across the HCV genotypes (*P* < 0.001) (Table 2). Patients with genotype 1a were more often F0 while genotype 1b infected patients were more likely to be classified as having F1 or F2 fibrosis. No other specific pattern was observed for the other subtypes.

### Phylogenetic analysis

**Fig. 3.**
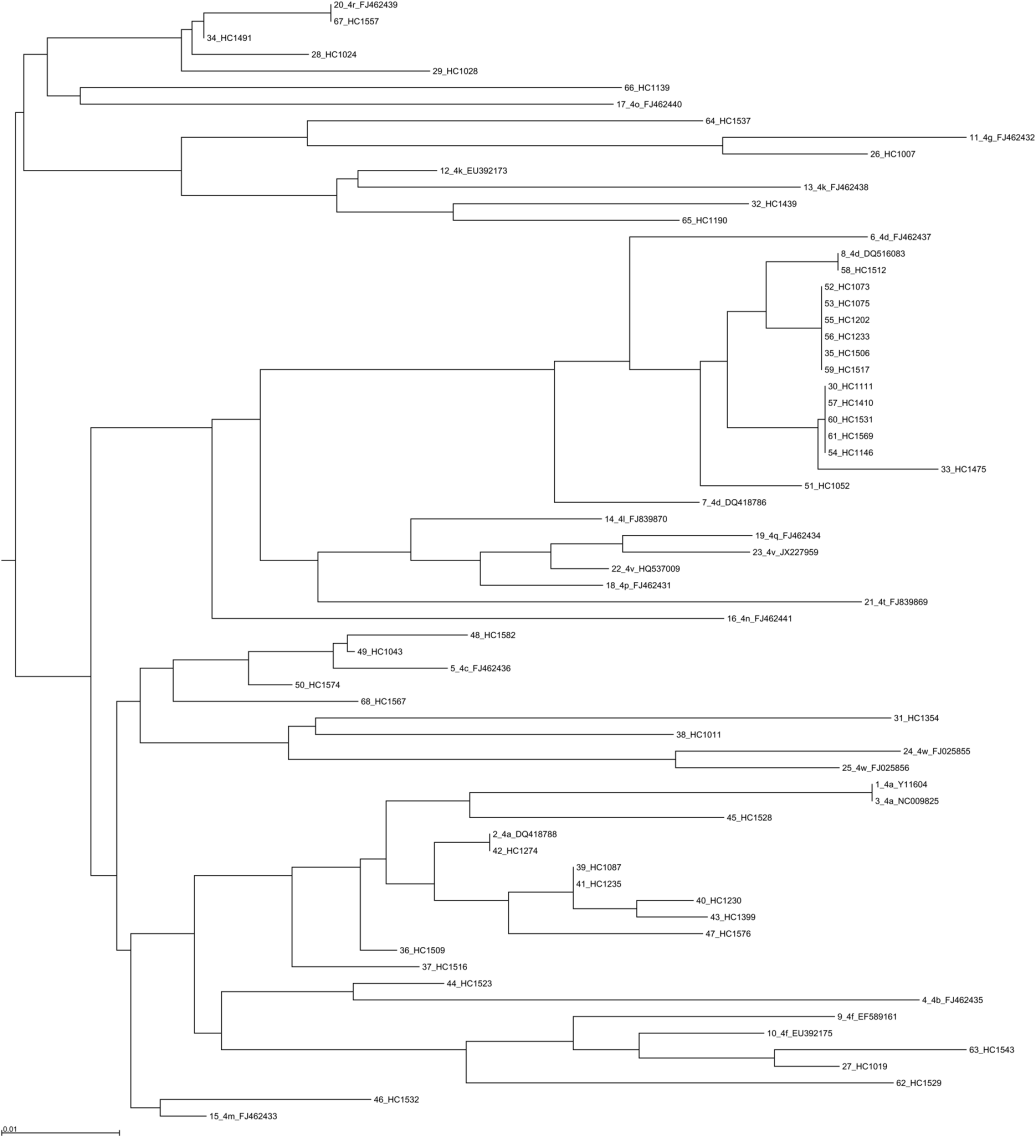
Phylogenetic tree based on NS5B sequences from 43 patients with 25 references for genotype 4. References are indicated in bold in the format: genotype.subtype_ID of isolate_GenBank assession number. The scale bars indicate the nucleotide substitutions per site. Analysis was performed using MAFFT software version 7.

Phylogenetic analysis was performed using 355 sequences: 330 sequences for the NS5B region (Fig. 3 and Supplementary Figs 4 and 5), 28 sequences for the NS3 region and 6 sequences for the core coding region. Some discrepancies were found between the genotype or subtype initially determined locally and that found using centralized phylogenetic analysis. One sample classified as genotype 3a by the initial laboratory was eventually identified as 2a by phylogenetic analysis (sample HC1525). Four samples (HC1582, HC1523, HC1537 and HC1011) initially determined as genotype 4a were subsequently identified as 4c, 4f, 4m and 4w, respectively (Fig. 3). One sample (HC1035) classified 2d by the local laboratory was assessed as a non-subtypable genotype 2 in the absence of robust clustering with a known genotype 2 sequence. Phylogenetic analysis of 387 NS5B sequences also allowed us to identify the subtype for 23 initially non-subtyped cases (2 for genotype 1, 4 for genotype 2, 5 for genotype 3 and 12 sequences for genotype 4).

Phylogenetic analyses did not reveal any particular clustering of the French sequences either in the international database, or among themselves.

## Discussion

In this French nationwide multicentre study, we determined the HCV genotype distribution in a large population of treatment-naïve HCV-infected patients with no or low-stage fibrosis. Genotype 1b was the most common genotype (27.2%), found in almost all age groups, but more frequently in patients aged over 60 than in younger patients and in women as previously described [19]. In the previous GEMHEP study conducted in 2005, genotypes 1b, 3 and 1a were the most frequent (27.6%, 21.1% and 18.6%, respectively) [20]. In the present study, a shift in HCV genotype occurrence was observed in treatment-naïve patients: genotype 1a becoming more frequent (to 25.7%), while a decrease in frequency was observed for genotype 3 (to 14.9%). A similar pattern of occurrence was seen in the GEHEP 005 Study of Spanish HCV viraemic patients with genotype 1b found in 41.3% of the patients and genotype 1a and 3 in respectively 24.9% and 19.6% [19]. One possible explanation for this increase in genotype 1a prevalence could be that it concerns mostly recent infections. Indeed, patients with subtype 1a were younger (mean 47 years) than those infected with genotype 1b (mean 53 years) and tended to have a less advanced liver disease with mild to moderate fibrosis. Genotype 1a was more frequent in patients with fibrosis stage F0 (28%), compared to genotype 1b, which was more frequent in patients with fibrosis stage F1 and F2 (28% and 29%, respectively). Our study was not designed to document the date of contamination and the only variable available was the patient's age. A better assessment of infection duration would have been an asset so as to better explore the difference between 1a and 1b infected patients. Viral load variations according to the genotypes are difficult to interpret. Higher HCV viral load with genotype 1a and lower with genotype 3 were already described without a clear explanation [21, 22].

Genotype 4 appears to be increasing in prevalence (13.8% here, compared with 9.2% and 9% in the Polaris and previous GEMHEP studies, respectively) [2, 20]. This, along with the low median age (46 ± 13) confirms the results of the Spanish GEHEP 005 study that suggested a recent introduction of genotype 4 into southern Europe [19]. Moreover, we found a large number of ‘non-a non-d’ genotype 4 (7.4% of the total and 53.8% of genotype 4) in patients originating from the Congo and Cameroon (6/6 of known origin) whereas those infected with subtypes a or d originated mainly from France (14/20 of known origin).

Shifts in population characteristics and hence in the prevalence of genotype 4 subtypes have potential consequences for public health. A recently published global analysis of HCV genotypes infecting 12 615 patients included in 67 clinical studies showed that the ‘non-a non-d’ genotype 4 represented only 0.55% of the population studied (14% of genotype 4) [23]. These subtypes may respond differently from genotype 4a or 4d to DAA combinations [24]. We note that genotype 4 ‘non-a non-d’ subtypes present in the French HCV-infected population are currently under-represented in clinical studies; therefore, no clear therapeutic recommendation exists for them. Such information is important if the current DAA strategy is to be used worldwide with the ambitious goal of eradicating HCV by the year 2030. The unexpected poor response of specific subtypes to DAA may lead to the selection of resistant variants, more difficult to eliminate.

Our study highlighted that (i) data from clinical studies lacks some HCV subtypes that are prevalent in France and even more prevalent in other parts of the world and (ii) only 17% of genotype 1a infected patients had a viral load <8 00 000 IU/ml and the rest would, therefore, be eligible for grazoprevir/elbasvir therapy as indicated in French recommendations [7]. Moreover, despite the arrival of theoretically pan-genotypic therapeutic combinations, differences in activity seem to persist, such as the lower efficacy of the glecaprevir/pibrentasvir combination on previously treated genotype 3 or of sofosbuvir/velpatasvir/voxilaprevir for 8 weeks on genotype 1a [25–27]. Precise pre-treatment knowledge of the viral subtype could thus be an important parameter in the therapeutic management of HCV infection and for the future goal of eradication of this infection.

While this study was not designed to allow a follow-up of the patients and the achieved SVR rates when treated, data were available for one centre. In this particular centre, 67 patients were included in the present study and 43 (64.2%) were treated between 2016 and 2019. Among those 43 patients, at least 27 (62.8%) achieved SVR.

One limitation of this study was the diversity of HCV genotyping assays and their interpretation, as shown by the number of discordant results due to variations in the sequences purchased by participating laboratories. These differences could be due to the use of different reference sequence databases. In 23 cases, the complementary phylogenetic analysis allowed the classification of unknown subtypes. Likewise, the use of a complete and consensual sequence database is crucial to correctly interpret HCV subtypes. The identification of a suspect NS5B sequence and its confirmation using a near-full-length genome sequencing approach, revealing a recombinant form of HCV, was only possible thanks to the introduction of RF2k/1b sequences into the database. The detection of other recombinant forms may be missed by short fragment sequencing, the absence of a reference recombinant sequence in the database or the use of a 5′UTR/Core-based Line probe assay [28]. We chose not to ask for detailed clinical data from each participating laboratory in order to include as many samples as possible without organisational restrictions. Furthermore, inclusions were deliberately restricted so as to focus on untreated patients with no or moderate fibrosis. This study, while based on a relatively short period, does, however, reflect the HCV infection spread in the French population by sampling patients from a large number of centres.

In conclusion, our study precisely describes the characteristics of HCV genotypes infecting patients who will soon benefit from expanded treatment indications recently recommended by the French government. Genotype 1b and 1a are currently the most frequent viral strains in treatment-naïve patients with mild fibrosis stages. Nevertheless, ‘non-a non-d’ genotype 4 is becoming increasingly frequent in this population and more clinical data on the efficacy of direct antiviral drugs on these emergent subtypes is needed.

## Supplementary material

For supplementary material accompanying this paper visit https://doi.org/10.1017/S0950268819001225. click here to view supplementary material
