# Supplementary figures and images for: Hepatitis C subtype distribution in chronically infected patients with mild liver fibrosis in France: the GEMHEP study
Source: Epidemiol Infect. 2019 Jul 10;147:e234. doi: 10.1017/S0950268819001225 (PMC6625182; doi:10.1017/S0950268819001225)

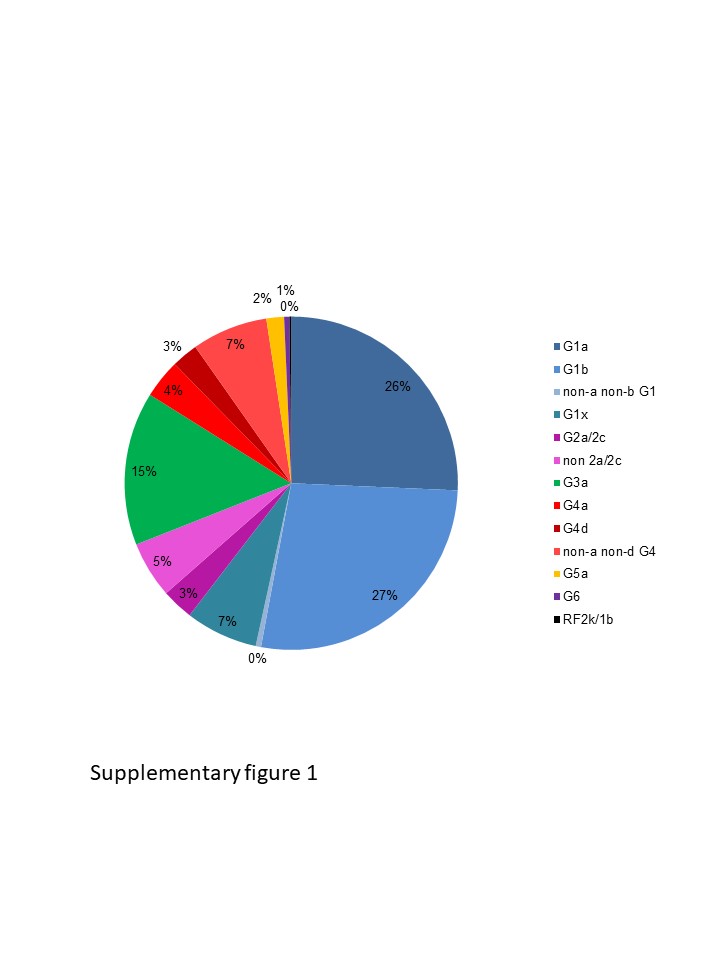

Supplement: Supplementary file 1 [file S0950268819001225sup001.zip › S0950268819001225sup001/Diapositive1.JPG]

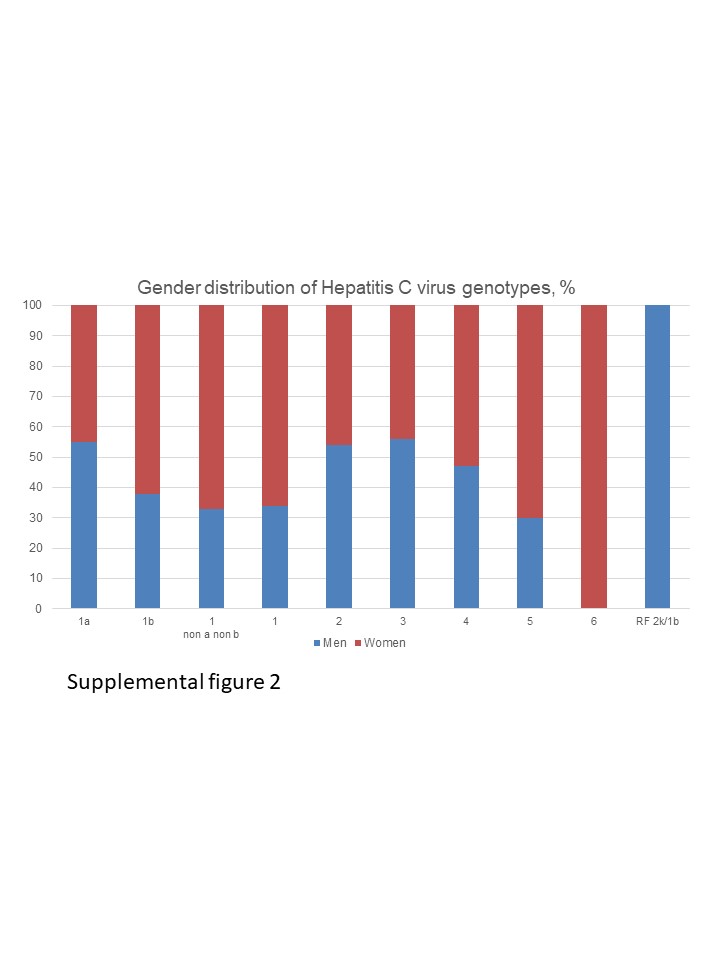

Supplement: Supplementary file 1 [file S0950268819001225sup001.zip › S0950268819001225sup001/Diapositive2.JPG]

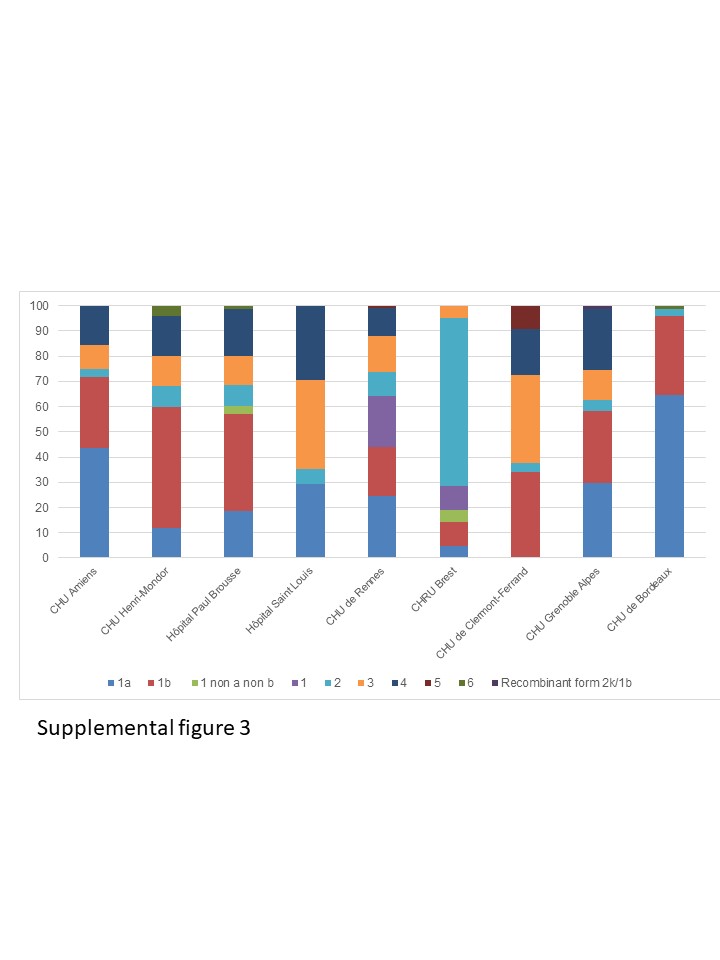

Supplement: Supplementary file 1 [file S0950268819001225sup001.zip › S0950268819001225sup001/Diapositive3.JPG]

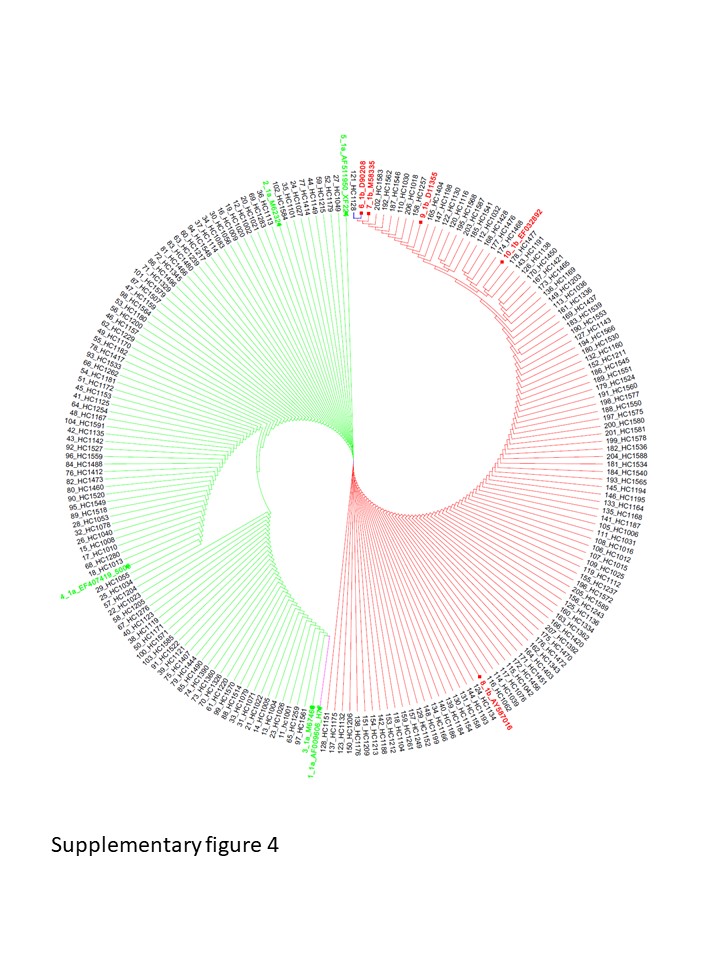

Supplement: Supplementary file 1 [file S0950268819001225sup001.zip › S0950268819001225sup001/Diapositive4.JPG]

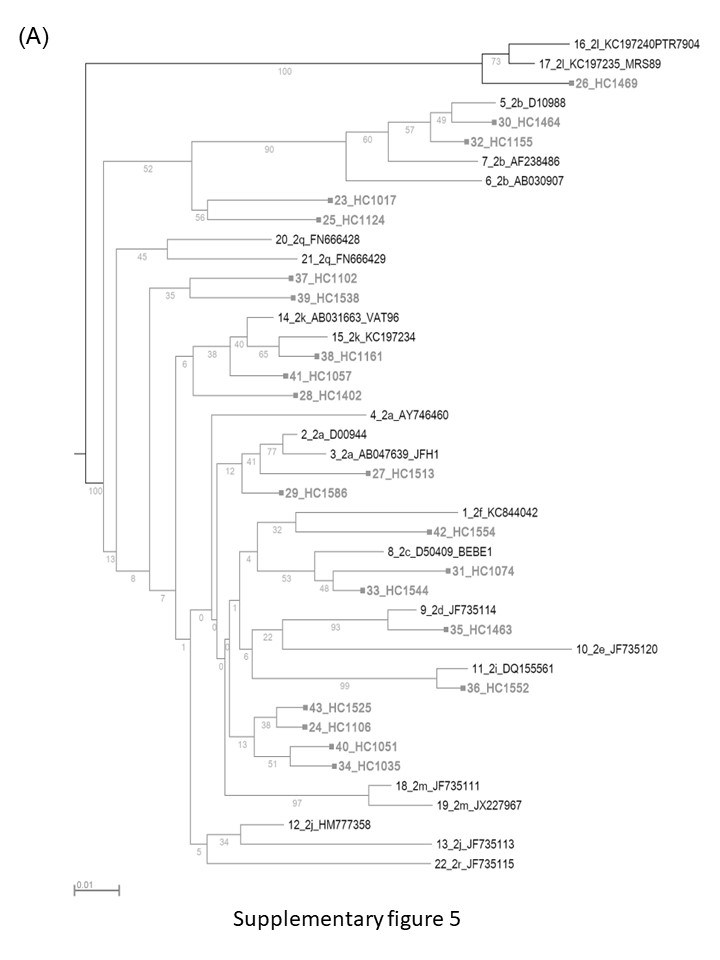

Supplement: Supplementary file 1 [file S0950268819001225sup001.zip › S0950268819001225sup001/figureS5A.jpg]

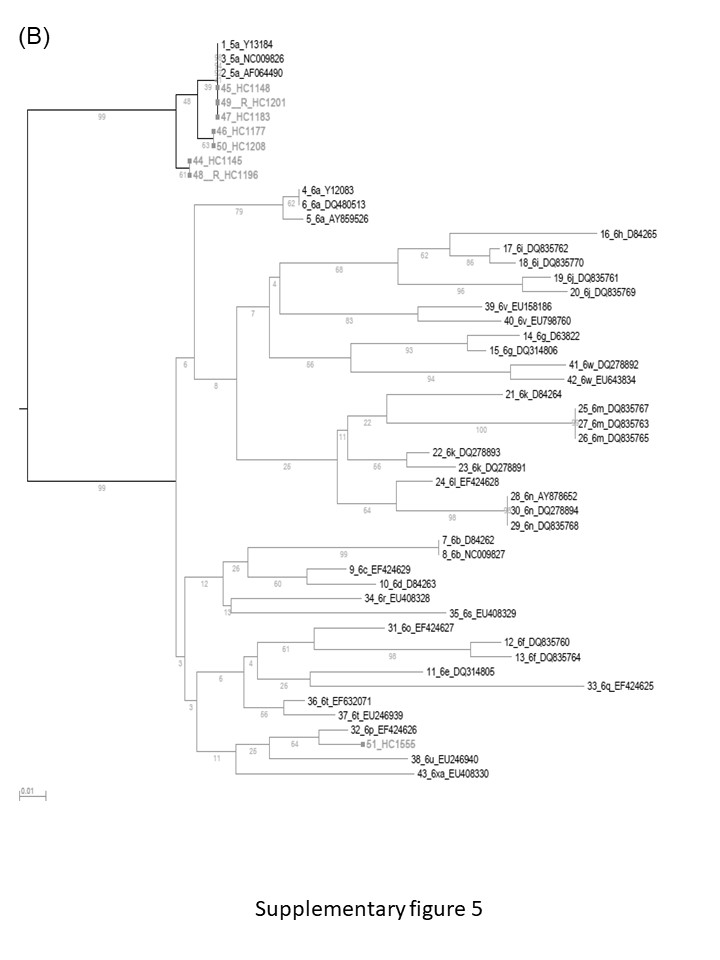

Supplement: Supplementary file 1 [file S0950268819001225sup001.zip › S0950268819001225sup001/figureS5B.jpg]
